# Beyond the Epidural: Reframing Analgesia for the Modern Oesophagectomy

**DOI:** 10.1111/ans.70685

**Published:** 2026-04-20

**Authors:** William Thomas Birkett, Geraldine Ooi, Bethany White, Hamish Shilton, Liang Low, Paul Cashin, Walston Reginald Martis

**Affiliations:** ^1^ Department of Anaesthesia and Perioperative Medicine Monash Health Melbourne Victoria Australia; ^2^ Department of Surgery, School of Clinical Sciences Monash University Melbourne Victoria Australia; ^3^ Department of Upper GI Surgery Monash Health Melbourne Victoria Australia; ^4^ Integrated Surgery Monash Health Melbourne Victoria Australia; ^5^ Department of Anaesthesia, Perioperative and Pain Medicine Peter MacCallum Cancer Centre Melbourne Victoria Australia; ^6^ Department of Critical Care University of Melbourne Melbourne Victoria Australia

**Keywords:** acute pain, enhanced recovery after surgery, epidural analgesia, oesophagectomy

## Abstract

A novel analgesic framework for minimally invasive oesophagectomy to facilitate functional recovery.

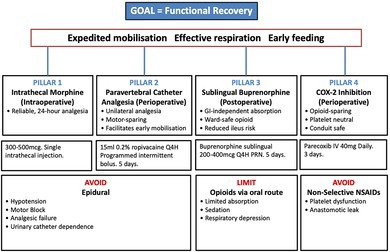

Oesophagectomy has undergone substantial evolution over recent decades. Once characterised by large thoraco‐abdominal incisions, this procedure has progressively transitioned through hybrid laparoscopic‐assisted techniques to totally minimally invasive (MIO) and increasingly robotic approaches. In parallel, Enhanced Recovery After Surgery (ERAS) principles have reshaped perioperative care [1], emphasising physiological stability and reduced length of stay [2]. Postoperative analgesia, however, has not evolved at the same pace.

Many centres continue to rely on thoracic epidural anaesthesia (TEA), once considered a ubiquitous gold standard for oesophagectomy [3], but increasingly misaligned with modern recovery goals. While TEA provides excellent analgesia at rest, thoracoscopic and laparoscopic incisions usually do not require such dense neuraxial blockade. Importantly, the physiological costs of this technique are significant. Blockade of thoracic sympathetic chains frequently results in hypotension [4], requiring vasopressors and intensive care admission. Extended lumbosacral spread can cause motor weakness and urinary catheter dependence [4], both impeding physiotherapy goals. Epidurals have also been shown to fail in just over 20% of cases [5], requiring specialist troubleshooting. All these factors introduce friction into contemporary evidence‐based [1, 2] ERAS pathways which demand expedited mobilisation, effective respiratory mechanics and early feeding [1], within a ward‐based environment.

Instead, a modern pathway should align analgesia with a broader enhanced‐recovery framework, as a single aspect of a multidisciplinary team. Achieving these goals, however, is challenging, as despite minimally invasive surgical techniques, oesophagectomy remains a painful and physiologically demanding thoracoabdominal procedure. Altered anatomy further limits the availability of the enteral route for medications. The optimal analgesia should therefore strike a careful balance: providing adequate and reliable pain control via limited routes to permit functional recovery, without excessive sedation, motor weakness, haemodynamic instability or ileus.

We propose a novel analgesic pathway aligned with modern MIO and perioperative care (Figure 1). This approach shifts away from routine TEA and instead centres on four pillars: intraoperative intrathecal morphine, perioperative paravertebral regional analgesia, postoperative sublingual opioid therapy and parenteral COX‐2 inhibition. This pathway is tailored to local Australian practice patterns, medication availability, and healthcare system constraints, while drawing on evidence from contemporary abdominal and thoracic surgery demonstrating the safety and efficacy of non‐epidural strategies [3, 4].

**FIGURE 1:**
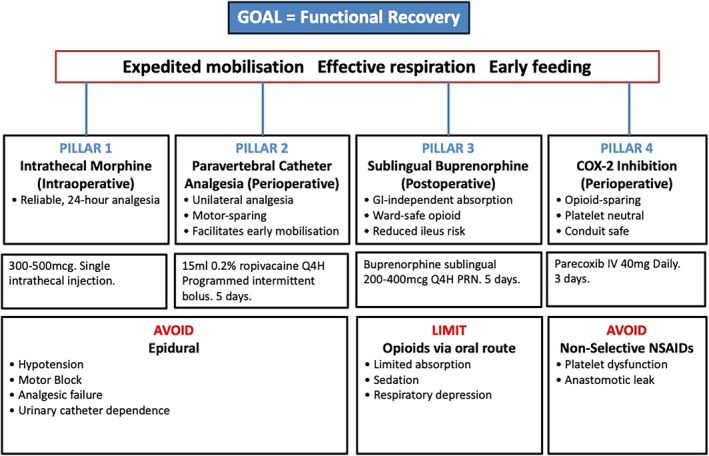
Analgesic framework for minimally invasive oesophagectomy. Abbreviations: COX, cyclooxygenase; GI, gastrointestinal; IV, intravenous; NSAID, non‐steroidal anti‐inflammatory drug; PRN, as needed; TDS, three times daily; Q4H, every 4 h; Q6H, every 6 h.

## 1 Intrathecal Morphine

The first pillar, the cornerstone, is intraoperative intrathecal morphine, and it serves as a transitional opioid strategy in the immediate postoperative period. Administered as a single injection prior to induction, it provides up to 24 h of analgesia, functioning as a short‐term bridge without the haemodynamic consequences of TEA. Multiple studies across colorectal, hepatobiliary and urological surgery demonstrate comparable analgesia between intrathecal morphine and epidural techniques, with reduced hypotension, fewer urinary catheters, and lower rates of analgesic failure [6, 7]. The perceived risk of delayed respiratory depression is mitigated by routine postoperative monitoring of MIO in the high dependency unit. Intrathecal buprenorphine represents a potential future evolution, allowing continuity of opioid therapy with a lower incidence of respiratory adverse effects [8].

## 2 Paravertebral Catheters (PVC)

Paravertebral regional analgesia via a catheter technique provides effective thoracic analgesia without the physiological burden of neuraxial blockade. Unilateral segmental analgesia avoids the sympathetic blockade and hypotension associated with epidurals [6], facilitating earlier discharge from intensive care. Motor function remains intact and urinary catheters can be removed earlier, enabling physiotherapy. In addition to these physiological advantages, PVCs provide comparable chest wall analgesia. The PEPMEN study demonstrated equivalent pain control between PVCs and thoracic epidurals following MIO [9], consistent with thoracic surgery literature supporting PVC use after thoracotomy [10].

Paravertebral catheters are placed after the completion of surgical resection under direct thoracoscopic vision alongside the sympathetic chain, and tunnelled using a malleable introducer to reduce dislodgement during postoperative patient mobilisation. Alternatively, the catheter can be placed under ultrasound guidance. We advocate a 0.2% ropivacaine programmed intermittent bolus regimen, delivering a four‐hourly 15 mL bolus, which provides superior analgesia compared with continuous infusion [11]. Ropivacaine is selected for its favourable safety profile over bupivacaine [12]. Catheters are removed by protocol on postoperative day five.

An additional single‐shot chest wall block prior to surgical incision provides further advantages. A pre‐emptive paravertebral or erector spinae plane block provides opioid‐sparing analgesia but theoretically may also reduce the risk of developing persistent post‐surgical chest wall pain [13], a significant long‐term symptom burden in oesophageal cancer survivors [14].

## 3 Sublingual Buprenorphine

Systemic opioid therapy remains an important component of postoperative analgesia; however, oesophagectomy presents unique challenges to conventional opioid delivery. The presence of an oesophageal anastomosis limits oral administration, and jejunostomy tubes are increasingly avoided because of their associated complications [15]. Narrow‐bore nasojejunal tubes are also prone to blockage, risking interruption to enteral nutrition. Intravenous opioid strategies may be associated with ileus, respiratory depression, and delirium, and necessitate physical tethering to intravenous lines and infusion devices, which can act as a barrier to early mobilisation and recovery [1].

Sublingual buprenorphine offers a pragmatic alternative when supplemental opioid is required. Its absorption is independent of gastrointestinal function and does not rely on enteral access. It has a predictable onset, a prolonged duration of action of approximately 6 h [16], and demonstrated efficacy following major abdominal surgery [17]. Compared with transdermal administration, the sublingual route provides superior reliability and safety, as transdermal absorption is influenced by skin blood flow and is characterised by a delayed onset related to the time required to establish a subcutaneous drug depot.

As an atypical opioid, buprenorphine acts as a partial μ‐opioid receptor agonist, conferring a ceiling effect on respiratory depression and reduced risk of opioid‐induced ventilatory impairment (OIVI) [16]. Used intermittently on a pro re nata (PRN) basis, it offers flexible supplemental analgesia while preserving the opioid‐sparing intent of the multimodal pathway. This approach has a more favourable safety profile than transdermal or sustained‐release formulations and is consistent with national opioid stewardship standards [18]. The 200 μg sublingual wafer provides analgesia equivalent to approximately 5 mg of oral oxycodone [19].

## 4 COX‐2 Inhibition

Perioperative non‐steroidal anti‐inflammatory drugs form a key component of multimodal analgesia. Parecoxib, an intravenous COX‐2 inhibitor available in Australia and New Zealand, reduces opioid requirements by up to 28% [20]. Unlike non‐selective NSAIDs, COX‐2 inhibitors have minimal gastric mucosal effects, reducing risk to the gastric conduit, and preserve platelet function. Concerns regarding NSAIDs and anastomotic leak arise largely from colorectal data and are confined to non‐selective agents; there is no demonstrated increased risk with parecoxib [21]. We utilise once‐daily intravenous dosing of 40 mg to provide effective analgesia without compromising anastomotic integrity.

As oesophagectomy continues to evolve toward minimally‐invasive techniques, analgesic strategies must evolve in parallel. Persisting with epidural‐centric models may limit the full advantages offered by modern minimally invasive oesophagectomy and ERAS pathways. We have proposed a recovery‐focused analgesic framework, integrating intrathecal morphine, paravertebral regional techniques, PRN sublingual opioid therapy, and selective anti‐inflammatory analgesia, that better aligns with contemporary perioperative goals. This framework has not yet been evaluated in a dedicated randomised trial in oesophagectomy and should therefore be regarded as a pragmatic, evidence‐informed proposal requiring prospective validation. Reframing analgesia as a facilitator of recovery rather than an endpoint represents a necessary step in the modernisation of oesophagectomy care.

## Data Availability

Data sharing not applicable to this article as no datasets were generated or analysed during the current study.
